# Social behaviour and transmission of lameness in a flock of ewes and lambs

**DOI:** 10.3389/fvets.2022.1027020

**Published:** 2022-12-01

**Authors:** Katharine E. Lewis, Emily Price, Darren P. Croft, Joss Langford, Laura Ozella, Ciro Cattuto, Laura E. Green

**Affiliations:** ^1^School of Life Sciences, University of Warwick, Coventry, United Kingdom; ^2^Centre for Research in Animal Behaviour, University of Exeter, Exeter, United Kingdom; ^3^Activinsights Ltd, Cambridge, United Kingdom; ^4^Department of Veterinary Science, University of Turin, Turin, Italy; ^5^Institute for Scientific Interchange (ISI) Foundation, Turin, Italy; ^6^Department of Computer Science, University of Turin, Turin, Italy; ^7^Institute of Microbiology and Infection, University of Birmingham, Birmingham, United Kingdom

**Keywords:** sheep, footrot, transmission, proximity sensor, social network, network based diffusion analysis

## Abstract

**Introduction:**

Sheep have heterogenous social connections that influence transmission of some infectious diseases. Footrot is one of the top five globally important diseases of sheep, it is caused by *Dichelobacter nodosus* and transmits between sheep when infectious feet contaminate surfaces, e.g., pasture. Surfaces remain infectious for a few minutes to a few days, depending on surface moisture levels. Susceptible sheep in close social contact with infectious sheep might be at risk of becoming infected because they are likely to step onto infectious footprints, particularly dams and lambs, as they cluster together.

**Methods:**

High resolution proximity sensors were deployed on 40 ewes and their 54 lambs aged 5–27 days, in a flock with endemic footrot in Devon, UK for 13 days. Sheep locomotion was scored daily by using a 0–6 integer scale. Sheep were defined lame when their locomotion score (LS) was ≥2, and a case of lameness was defined as LS ≥2 for ≥2 days.

**Results:**

Thirty-two sheep (19 ewes, 9 single, and 4 twin lambs) became lame during the study, while 14 (5 ewes, 5 single, and 4 twin lambs) were lame initially. These 46 sheep were from 29 family groups, 14 families had >1 lame sheep, and transmission from ewes to lambs was bidirectional. At least 15% of new cases of footrot were from within family transmission; the occurrence of lameness was higher in single than twin lambs. At least 4% of transmission was due to close contact across the flock. Most close contact occurred within families. Single and twin lambs spent 1.5 and 0.9 hours/day with their dams, respectively, and twin lambs spent 3.7 hours/day together. Non-family sheep spent only 0.03 hours/day in contact. Lame single lambs and ewes spent less time with non-family sheep, and lame twin lambs spent less time with family sheep.

**Discussion:**

We conclude that most transmission of lameness is not attributable to close contact. However, in ewes with young lambs, some transmission occurs within families and is likely due to time spent in close contact, since single lambs spent more time with their dam than twin lambs and were more likely to become lame.

## Introduction

Footrot is one of the top five globally important diseases of sheep and causes lameness, poor welfare and production losses (1–4). Footrot is caused by *Dichelobacter nodosus*. Diseased feet are infectious for 3 days to > 10 weeks, depending on the time to recovery (5). Diseased feet deposit *D. nodosus* on surfaces such as pasture where it is detected in only some samples of soil from areas where sheep spend considerable time, e.g., around feed and water points, and from areas where sheep are only transiently present (5). This distribution of detection in both high and low use areas of a pasture is explained by the nature of *D. nodosus—D. nodosus* cannot replicate off-host (5, 6) and only survives in the environment for a few minutes to a few days; survival time increases in moist environments and pasture can be persistently contaminated in wet weather (5, 7).

The incidence of footrot increases in flocks of ewes with young lambs. This has been attributed to high stocking densities, moist weather conditions, and susceptible newborn lambs (8). The incidence of footrot also clusters within families [dam and lamb(s)] (9). Within family clusters could be explained by genetic susceptibility to footrot, however, heritable resistance is low (h = 0.1) (10) and it is possible that close proximity explains at least some transmission (indirect *via* pasture) of footrot within families (11–15). Dams and their lambs spend considerable time together and the majority of physical contacts within family are close and prolonged, e.g., suckling, whilst contact between non-family ewes and lambs tend to be more distant and brief, e.g., sniffing (14).

Since social affinity in sheep is highly correlated with spatial proximity (16), proximity sensors are a suitable tool to assess their social behavior and have been used previously in studies assessing contact patterns in sheep (17–19). Proximity sensors collect high-resolution, continuous data of spatial co-location between two animals, and these types of biologging technologies are particularly useful to investigate transmission of disease because large volumes of data are collected continuously for each animal without the need for continuous observation and without disturbing the animals.

Analysis of data on disease transmission between pairs of animals cannot be carried out using traditional statistical tools because paired data are inherently related and defy the rules of independence, i.e., sheep A and B in contact at time t is the same data as sheep B and A in contact at time t (20). Network-based diffusion analysis (NBDA) can use association probabilities that account for the behavior of both sheep A and sheep B directly, to investigate whether transmission of a disease occurs through a social process (21), by determining if the spread of disease follows the connections in a social network (22). So for footrot, the connection of a lame sheep with a non-lame sheep could increase the risk of transmission of disease.

The aim of our study was to use proximity sensors and NBDA to determine whether family spatial co-location contributes to the spread of footrot between ewes and lambs. We used data from Ozella et al. (15) together with visual observations of lameness from the same flock at the same time.

## Materials and methods

Ethical approval was granted by the University of Exeter (eCLESPsy000541).

### The study location, population, pasture management and climate

The study took place from 1st−15th October 2019 on a farm with permanent grass pasture in the Blackdown Hills, Devon, United Kingdom. The study population was a commercial flock of 50 pedigree Poll Dorset ewes with 68 lambs, sired by 5 rams. The breeding cycle on the study farm [described fully in (19)] was typical for Poll Dorsets, with mating in mid-April and parturition from September to mid-October. Poll Dorset ewes are sexually active for most of the year and produce lambs from August–May in the Northern Hemisphere.

Each ewe was identified with a unique number with livestock marker paint, and her lambs were marked with that same number, with the larger twin in a litter differentiated by a paint dot on the head. On day 0 (October 1st), all sheep were moved onto a field which had not been grazed by sheep for at least 4 weeks to ensure that the pasture was free from *D. nodosus* and so not a source of infection of *D. nodosus* (5–7). The field was surrounded by large hedgerows on all sides, with the water trough positioned at one side, and was managed by strip grazing using an electric fence. Initially the flock had access to 0.69 hectares (ha) of pasture, this was increased to 1.34 ha after 4 days, then to 1.98 ha after a further 4 days. Meteorological data were collected daily using a Davis Vantage Pro2 Plus weather station (Supplementary Table 1) and summarized into two climatic indexes—the mean daily temperature-humidity index (THI, °C), which combines temperature and humidity (23) and the mean daily wind-chill index (WCI, °C) which combines wind speed and temperature (24).

### Proximity sensor deployment

The proximity sensing platform was designed by the SocioPatterns collaboration consortium (http://www.sociopatterns.org/). The sensors have a bidirectional radio interface and transmit packets carrying a unique identifier as a data payload, which is received by nearby sensors. The exchange of packets is used to measure spatial proximity by attenuation, i.e., the difference between the received and transmitted power (25). An attenuation threshold of −75 dBm was used to detect sheep co-located within 1–1.5 m. Information on previous calibration and validation of the sensing system are in Fielding et al. (26). Based on the calibration and validation work of Fielding et al. (26) and a number of studies with sheep (15, 19), we have confidence that an attenuation threshold of −75 dBm characterizes proximity between on-sheep sensors of within 1–1.5 m (approximately the body length of an adult sheep). Because the maximum distance detected between two sensors is 5 m (26), we can state that the above mentioned attenuation threshold characterizes proximity never exceeding 5 m. A co-location occurred when at least one radio packet was exchanged which exceeded the attenuation threshold. The duration of a co-location was the number of consecutive 20 s time intervals in which radio packets were exchanged.

The proximity sensors used in this study have been used in other social network studies on animals (15, 19, 26, 27). The sensors weigh ~6 g (sensor ~ 2.7 g, lithium coin battery ~ 3 g), and have a memory of ~1,000 h of contact events and a battery life of ~25 days. Sensors were put on the ewes on freely rotating neck collars and on lambs on adjustable body harnesses that were checked daily and enlarged as they grew. The total weight of the equipment was ~100 g, < 5% of an animal's body weight as recommended (28, 29).

Proximity sensors were fitted to all 50 ewes and 65/68 lambs; three were considered too small to wear the device. Not all devices collected data for the whole study. We excluded whole family groups if one family member was not tagged, or experienced device issues [see (15)], leaving 40 ewes and 54 lambs with complete data.

### Occurrence of footrot, locomotion scoring and foot lesion scoring

Most sheep with footrot are lame (30), consequently lameness was used as a proxy for footrot and it was assumed that only lame sheep were infectious. Ewes and lambs had been acclimatized to locomotion scoring by KL walking through the flock and scoring locomotion each week throughout September 2019. For the study period (1st−15th October), the locomotion of all ewes and lambs was assessed once each day by KL using a validated locomotion scoring system (31) (Supplementary Table 2). Scores were recorded on paper and entered manually into Microsoft Excel (32). An episode of lameness was defined as a sheep with a locomotion score ≥2 on ≥2 days. The daily and cumulative prevalence of lameness in ewes, single and twin lambs were calculated.

Interdigital dermatitis (ID), a presentation of footrot (33) was observed in sheep during the study. Nine ewes and 10 lambs, identified by the farmer, were treated by spraying the interdigital skin of all four feet with topical antibiotic. No other infectious causes of lameness were observed. The feet of all ewes and lambs were inspected on the last day of the study and footrot lesions were scored (34) (Supplementary Table 3) by EN, who was blind to the locomotion scores of the sheep. Other foot lesions (white line disease, fibroma, and granuloma) were identified and recorded using classical definitions (35).

### Summary statistics and network visualization

All analysis was done using RStudio v4.0.3. The social network was visualized using *igraph* (36) and *ggnetwork* (37). Sheep have clear and consistent diurnal patterns with periods of inactivity during the night (38) but sleep in short bouts (39) therefore the start of each 24-h period can be chosen arbitrarily and was selected as midnight for the study because that provided 13, 24-h periods. The day of deployment and removal of sensors was not included in the analysis of data. The time sheep spent with other sheep per day was calculated by summing the 20-s contact periods over a 24-h period for within family, and out of family contacts.

The probability of association between pairs of sheep was measured using dyadic association indexes to estimate the proportion of time pairs spent in close contact. The index was calculated as in Ozella et al. (15) with the formula:


(1) $$\begin{array}{l} AI=\;\frac{x_{ab}}{x_{ab}\;+\;x_{a}+\;x_{b}} \end{array}$$

where the association index (AI) equals the number of 20-s sampling periods *x*, with individuals *a* and *b* in contact, divided by the number of sampling periods where individuals *a* and *b* were in contact, plus the number of sampling periods where *a* was detected without *b*, and the number of sampling periods where *b* was detected without *a*. The index ranges from 0 (two individuals were never observed together) to 1 (two individuals were always observed together) consequently, the higher the value of the index, the greater the contact between a pair of sheep.

### Multi-network network-based diffusion analysis to investigate the role of social networks in transmission of footrot

Multi-network NBDA was carried out using the *NBDA* R package (40) to quantify the relative importance of different social networks in transmission of *D. nodosus*. Within a multi-network NBDA, social networks are used as predictor variables which represent hypotheses about possible transmission pathways of a trait. In our analysis social transmission of footrot occurred if a sheep became lame following contact with a lame sheep, and asocial transmission of footrot occurred if a sheep became lame without contact with a lame sheep. The Akaike weight was used to rank models, using the probability that the model was the best Kullback-Leibler model in the set, given the dataset and candidate models (41).

The basic NBDA model (40) is fitted by maximum likelihood and is expressed as:


(2) $$\begin{array}{l} \lambda_{i}\left(t\right)=\;\lambda_{o}\left(t\right)\left(1-z_{i}\left(t\right)\;\right)\;\left(s\sum_{j=1}^{N}a_{ij}z_{j}\left(t\right)+1\right) \end{array}$$

where λ_*i*_(*t*) is the rate at which individual *i* acquires the disease as a function of time *t*, λ*o*(*t*) is the baseline rate of disease acquisition, *z*_*i*_(*t*) is the “status” of individual *i* at time *t* (1 = diseased; 0 = naive), *N* is the number of individuals in the population and *a*_*ij*_ is a non-negative value indicating the connection strength from *j to i:* connection strength takes values from 0 = never associated to 1 = always associated in a social network, *s* is the key output parameter, it is the relative strength of social transmission of lameness to asocial transmission of lameness.

Multinetwork NBDA (42) expands this to:


(3) $$\begin{array}{l} \lambda_{i}\left(t\right)=\;\lambda_{o}\left(t\right)\left(1-z_{i}\left(t\right)\;\right)\left(e^{{\text{Γ}}_{i}\left(t\right)}\underset{n}{\sum }s_{n}\;\overset{N}{\underset{j=1}{\sum }}a_{n,ij}z_{j}\left(t\right)+e^{B_{i}\left(t\right)}\right) \end{array}$$

where *a*_*n, ij*_(*t*) is the connection strength from *j* to *i* in network *n* at time *t* (day of the study), and *sn* is the transmission rate per unit connection in network *n* relative to the rate of asocial transmission, and z is the status (0 or 1) of the individual. B_i_ is the sum of the coefficients of the effect of variables 1:*k* on asocial transmission multiplied by the value for individual i, and Γ_*i*_ is the sum of the coefficients of the effects of variables 1:*k* on social transmission multiplied by the value of variable *k* for individual *i*.

A summary of all candidate models tested are in Table 1. We detail below which predictors were used and why.

**Table 1:** Reference table for each model with the combination of individual-level variables and baseline rates of acquisition of lameness.

		**Type of transmission**			
**Social network hypothesis**	**Baseline rate of acquisition of lameness**	**Social and asocial**	**Social only**	**Asocial only**	**No predictor variables included**
H1: kinship network (family groups)	Constant	1	2	3	4
	Weibull	17	18	19	20
	Gamma	33	34	35	36
H2: Spatial proximity—all sheep	Constant	5	6	7	8
	Weibull	21	22	23	24
	Gamma	37	38	39	40
H3: Spatial proximity—non-family sheep	Constant	9	10	11	12
	Weibull	25	26	27	28
	Gamma	41	42	43	44
H4: Homogenous network	Constant	NA	13	14	15
	Weibull	NA	29	30	31
	Gamma	NA	45	46	47
No social network effect (all sheep independent)	Constant	–	–	16	–
	Weibull	–	–	32	–
	Gamma	–	–	48	–

^a^ Individual level predictors were dummy coded variables for whether a sheep was a lamb or not, or a twin or not.

^b^ NA indicates where a model could not be estimated due to convergence issues and was not given a model-reference number,—is used where parameters do not apply (i.e., models without a social network used as predictor, therefore social effects are not be estimated), H, hypothesis.

Cell entries are the model number.

There were four hypotheses on transmission pathways of *D. nodosus* through social networks in the flock tested, these were:

Hypothesis 1: there is an increased risk of transmission of footrot within families. To test this the connection strength between sheep i and j was set to 1 if there was a family relationship, and 0 for a non-family relationship. This hypothesis did not investigate spatial proximity, rather family networks.
Hypothesis 2: the time sheep are in close proximity to each other increases the risk of transmission of footrot. To test this, the connection strength between sheep i and j was set to their association probability from the dyadic association network over the whole study period.
Hypothesis 3: there is transmission of footrot only outside families. To test this, the model was set up as for hypothesis 2, but with connection strength set to 0 for family members, excluding contacts between sheep in the same family.
Hypothesis 4: all sheep have equal risk of becoming lame; footrot is not spread by close contact between sheep. To test this, the connection strength between all pairs of sheep was set to 1. If this homogenous network is favored over the network hypotheses 1–3, it would imply that all sheep have an equal risk of becoming lame.

Visualizations of the connection strength for each of these four hypotheses for example family groups are shown in Supplementary Figure 1.

Models were also run without inclusion of a social network predictor in order to determine if inclusion of a social network (hypothesis 1–4) improved model fit compared to use of individual-level predictors alone. Since it is plausible that the likelihood of whether a sheep becomes lame as a result of social or asocial transmission could be affected by whether the sheep is a ewe, single or twin, individual-level predictors (dummy coded predictors for whether a sheep was a lamb or not, or a twin or not) were investigated to see if they affected the baseline rate of acquisition or the social rate of acquisition of lameness, or both, as well as models without individual-level predictors. The exponential of the coefficient for an individual-level predictor gives a rate, where <1 is a decrease, and >1 is an increase.

In addition, three assumptions on the baseline rate of acquisition of lameness were tested (43), these were:

- (A) Constant: the rate of acquisition of lameness remained constant over time
- (B) Gamma distribution: a systematic increase or decrease in the rate of asocial acquisition of lameness over time
- (C) Weibull distribution: a varying rate of acquisition of lameness with an additional shape parameter κ, when x >1 increasing baseline rate, a constant baseline where κ =1 and a decreasing baseline where κ < 1.

Multi-model inferencing across the model sets for NBDA was used to calculate the median estimates of the parameters to provide robust inference about the strength of transmission through the different networks and the effects of predictor variables (40, 41). A lower limit calculation for the s parameter was performed for relevant models, using the profile likelihood function to search between 0 and the maximum likelihood estimate for s in each model. This is because NBDA can have more certainty about the lower limit of s than the upper limit because the profile likelihood is highly asymmetrical.

The proportion of cases solved by social transmission corresponding to the lower limit of *s* were calculated as in Hoppitt et al. (40):


(4) $$p_{social,\;e\;}=\;\frac{e^{{\text{Γ}}_{i}\left(t_{e}\right)s\;\sum_{j=1}^{N}a_{ij}(t_{e})z_{j}(t_{e})}}{e^{{\text{Γ}}_{i}\left(t_{e}\right)s\;\sum_{j=1}^{N}a_{ij}(t_{e})z_{j}\left(t_{e}\right)+\;e^{B_{i}(t_{e})}}}$$

Where *i* is the sheep that became lame during event *e* **(**the case of lameness), and *t*_*e*_ is the day of the study at which event *e* occurred. This is the predicted relative social transmission rate divided by the predicted total relative transmission rate for *i* at the time of transmission. The mean of p_social, e_ across all events is the estimated proportion of events that occurred by social transmission. All other parameters are as defined in Equations (2) and (3).

### Mixed effect models of associations between sheep time in contact with family, and non-family sheep

The time per day each sheep spent with family and non-family sheep was used as the outcome variable in linear mixed effects models to investigate the relationship between time spent in contact with sheep and potential drivers of social contact using *lme4* (44). Six models were run with ewes, single and twin lambs investigated separately because their contact patterns varied [Supplementary Table 4 and Ozella et al. (15)]. Random effects were included for day and sheep. Fixed effects were ewe age (years), lamb age (days) and lamb sex, and measured factors that could affect sheep behavior: whether a sheep was lame, whether sheep were gathered, pasture size, mean daily THI (°C) and mean daily WCI (°C).

The models took the form:


(5) $$\begin{array}{l} y_{ij}=\beta_{0}+\beta x_{j}+\beta x_{ij}+u_{ij}+e_{i} \end{array}$$

where y was the continuous outcome variable time per day with family, or time with non-family, β_0_ was the intercept, β*x*_*j*_ were the explanatory variables that varied by sheep and β*xx*_*ij*_ were the explanatory variables that varied by day. Residual variance estimates were included at sheep (*u*_*ij*_) and day (*e*_*i*_).

Multi-model inferencing (41) using rank by Akaike's Corrected Information Criterion (AICc) was used to account for model selection uncertainty and to calculate model-averaged coefficients and confidence intervals for fixed effects. Variable importance was calculated as the sum of the Akaike weights over all models that included the variable.

## Results

### Episodes of lameness over the study

A total of 46 sheep from 29 families [dam and lamb(s)] were lame during the study; 5 ewes, 5 single, and 4 twin lambs were lame at the start of the study and a further 19 ewes, 9 single, and 4 twin lambs became lame (Table 2), giving a total of 24 lame ewes, 14 singles, and 8 twins. In 14 families with >1 lame sheep, transmission of lameness between ewes and lambs was bidirectional. Individual daily locomotion scores are in Supplementary Figures 2, 3.

**Table 2:** Descriptive characteristics for the 40 ewes and their 54 lambs with 13 days of midnight-midnight contact data and complete locomotion records.

**Characteristic**	**Ewes (*****N*** = **40)**			**Lambs (*****N*** = **54)**		
**Categorical**	**Unit**	***N* ^a^**	**%**	**Unit**	***N* ^a^**	**%**
*Lameness*
Point prevalence	Day 0	5 (12.5)			5 (19.2)	4 (14.3)
Cumulative incidence	Day 1–13	19 (47.5)			9 (34.6)	4 (14.3)
Cumulative prevalence	Day 0–13	24 (60.0)			14 (53.8)	8 (28.6)
*Foot lesions*
Interdigital dermatitis	Day 14	12 (30.0)			9 (34.6)	11 (39.3)
Non-infectious^b^	Day 14	17 (42.5)			1 (3.8)	1 (3.6)
*Sheep characteristics*
Litter size	1	26	65.0	1	26	48.1
	2	14	35.0	2	28	51.9
Sex	Female	40	100.0	Female	27	50.0
	Male	–	–	Male	27	50.0
		**Mean**	**Range**		**Mean**	**Range**
Age^c^	Years	4	2–9	Days	15	5–27

a N, number.

b Non-infectious foot lesions include white line disease, fibroma, heel ulcers and other foot abnormalities (see Supplementary Table 5).

c Age, age at start of study period (01/10/2019).

Lameness was primarily caused by interdigital dermatitis (ID) (Table 2, Supplementary Table 5). On day 14 ID lesions were observed on 12 (30%) ewes, 9 (34.6%) single lambs, and 11 (39.3%) twin lambs, some lesions were very mild and not all sheep with ID were lame (Supplementary Table 5).

### Contacts between sheep recorded by the proximity sensors

**Figure 1:**
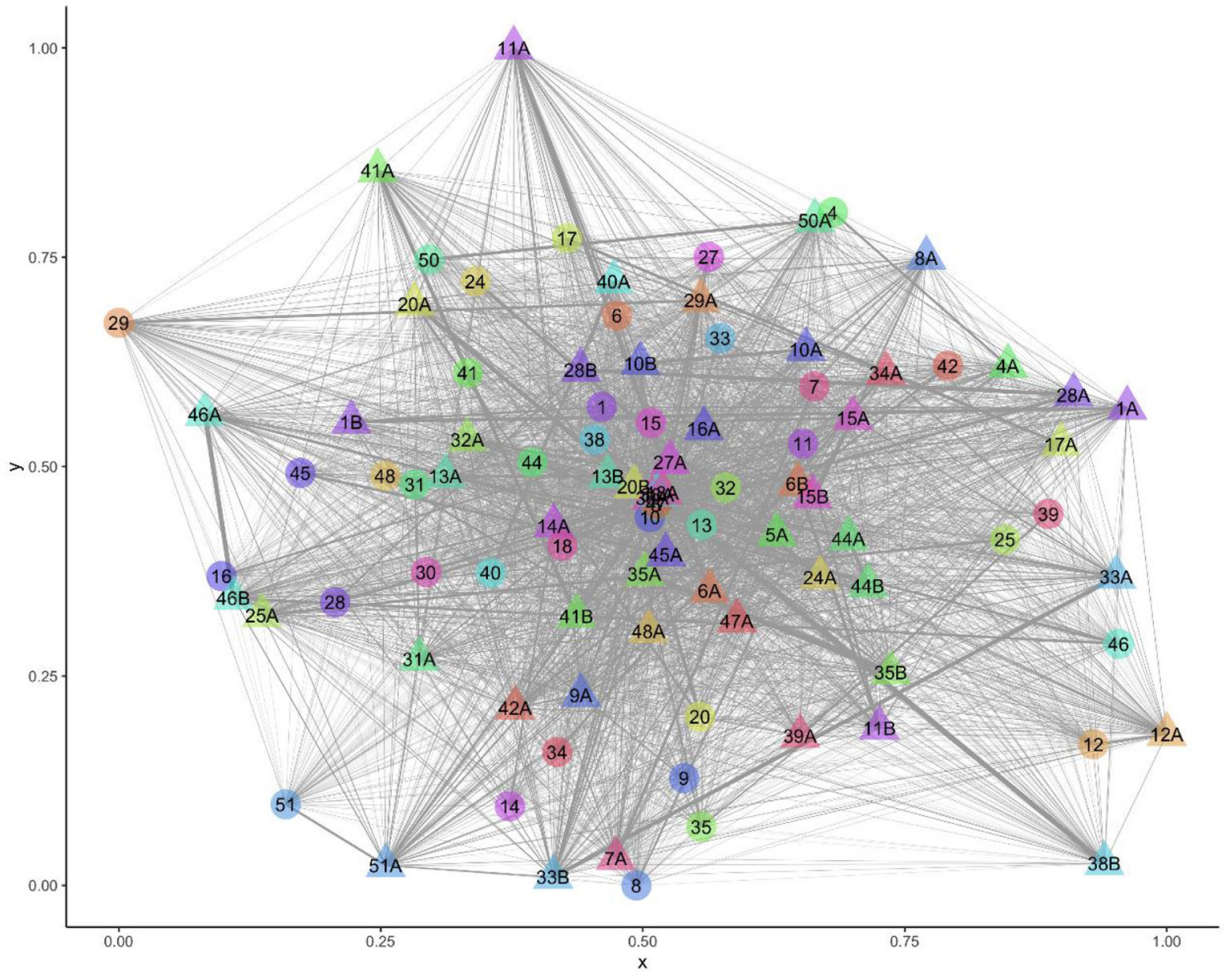
Visualization of the network over the entire study period with 94 nodes (sheep) and 4,358 edges (contacts). Ewe and lamb family groups have the same number, letter A denotes a lamb and B a twin to lamb A.

All sheep contacted almost all other sheep at some point in the study period (Figure 1). There were 216,054 contacts over the 13 days, there were 1,338–3,754 contact pairs per day, with 4,358 unique pairings over the 13 days, out of a possible 4,371. Overall, sheep spent an average of 5.27 hours/day [standard deviation (sd) = 3.26] with other sheep (Table 3). Twin lambs spent most time with other sheep (mean 8.55 hours/day), followed by single lambs (mean 5.43 hours/day), while ewes spent less time with other sheep (mean 2.87 hours/day): 64.2% of all contact time was within families. Single lambs spent 1.50 hours/day (sd = 1.05) with their dam, while twins spent 0.92 hours/day (sd = 0.78) with their dam and 3.69 hours/day (sd = 2.31) with each other (Table 3). The dyadic association indexes (AI) over the whole study period were strongest between twins (mean AI = 0.282), followed by single lambs and their dams (mean AI = 0.233), with weaker bonds between twin lambs and their dams (mean AI = 0.088) (Supplementary Table 4). The association between non-family sheep was extremely low (mean AI = 0.003), for single and twin lambs and 0.002 for ewes (Supplementary Table 4), and on average, sheep spent 0.03 hours/day with each non-family sheep.

**Table 3:** Descriptive statistics of hours/day over 13 days that sheep spent with other sheep: overall, within family and out of family for ewes, single and twin lambs.

**Sheep**		***N***	**Mean**	**Median**	**SD**	**Min**.	**Max**.
*Hours/day with all sheep*
All sheep		1222	5.27	4.50	3.26	0.00	17.29
Ewe		520	2.87	2.74	1.41	0.00	7.99
Single lamb		338	5.43	5.28	2.26	0.11	12.92
Twin lamb		364	8.55	8.28	3.02	1.74	17.29
*Hours/day with family*
Ewe		520	1.62	1.30	1.14	0.00	5.93
Single lamb		338	1.50	1.24	1.05	0.03	5.93
Twin lamb	(all family)	364	4.61	4.64	2.59	0.00	10.69
	(dam)	364	0.92	0.70	0.78	0.00	4.22
	(sibling)	364	3.69	3.52	2.31	0.00	9.62
*Hours/day with non-family*
Ewe		520	1.25	1.04	0.89	0.00	5.98
Single lamb		338	3.94	3.67	2.19	0.08	10.60
Twin lamb		364	3.94	3.52	2.56	0.14	15.44

^a^ N, number of sheep day observations, Mean, arithmetic mean, SD, standard deviation, Min, minimum, Max, max.

### The role of family and spatial proximity in spread of footrot: Multi-network network-based diffusion analysis

#### Model-averaged parameters and potential transmission pathways of *D. nodosus* through the flock

**Figure 2:**
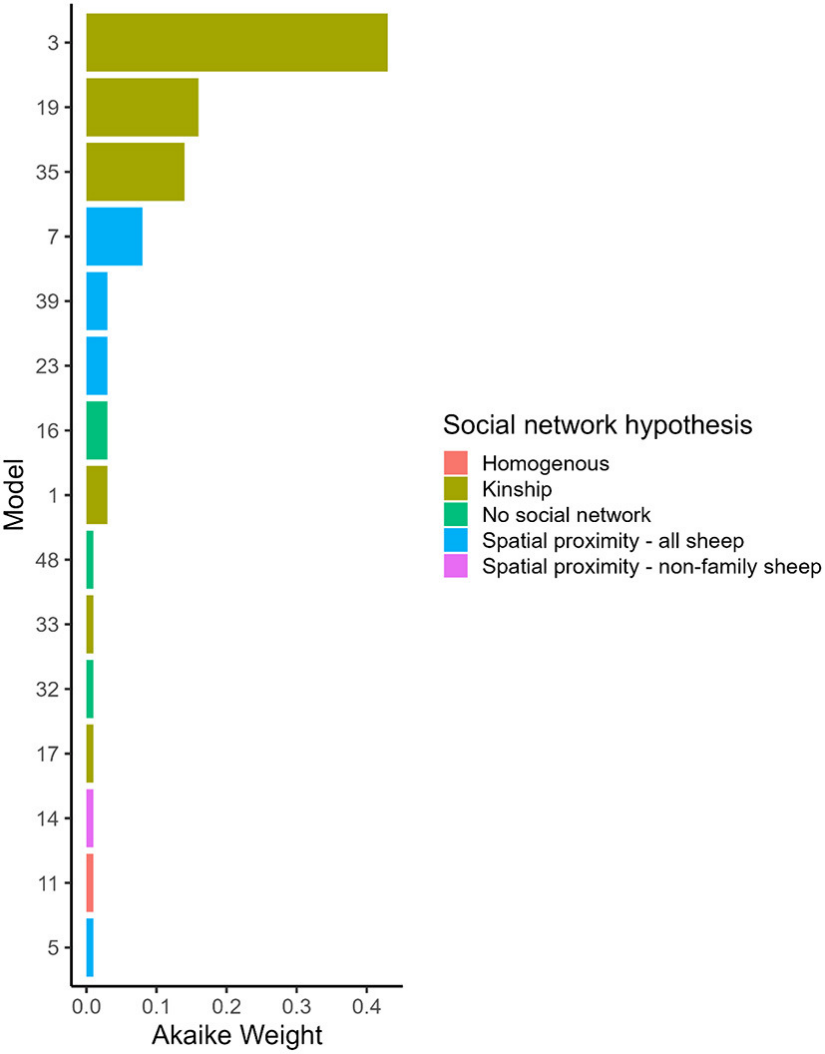
Akaike Weight for models with different social network predictors and combinations of variables affected social and asocial transmission. Models are ordered by Akaike Weight and are shown if the Akaike Weight was >0.00. 1. Full descriptions of the models are in Table 1 and the full set of Akaike Weights are in Table 4. Akaike Weight is the probability the model is the best Kullback-Leibler model in the set.

Hypothesis 1 (lameness is transmitted within families) was the most favored transmission pathway (Figure 2) and models where the family network was the social network predictor had ∑ *Akaike Weight*_*overall*_ = 0.78 (Table 4). Within the family network models, lambs became lame at 0.59 times the rate of ewes, and twin lambs became lame at a much slower rate, 2.70 × 10^−9^ times, than single lambs and ewes (Table 5). Models fitted best when the sheep predictor (whether a sheep was a ewe or lamb, and a twin or single) affected the rate of asocial transmission of lameness only (Table 4), which indicated ewes and lambs had different baseline rates for acquisition of asocially derived lameness. When this is accounted for (models 3, 19 and 35), at least 15% of cases were attributed to social transmission (Table 4).

**Table 4:** Lower confidence interval for the *s* parameter, the corresponding proportion of cases estimated to be by social transmission, and Akaike Weights for each model, ranked by overall Akaike Weight.

**Model^a^**	**LCI s**	**PST**	**ΔAICc**	**Akaike Weight_overall_**	**Akaike Weight_adjusted_**	**Akaike Weight_cumulative_**
**(H1) Kinship network (family groups)**						
3	0.13	0.15	0.00	0.43	0.55	0.55
19	0.13	0.15	2.02	0.16	0.20	0.76
35	0.13	0.15	2.26	0.14	0.18	0.94
1	0.00	0.14	5.27	0.03	0.04	0.98
17	0.00	0.14	7.87	0.01	0.01	0.99
33	0.00	0.14	8.09	0.01	0.01	1.00
4	0.00	0.00	11.79	0.00	0.00	1.00
20	0.00	0.00	13.23	0.00	0.00	1.00
36	0.00	0.00	13.42	0.00	0.00	1.00
2	0.00	0.00	15.23	0.00	0.00	1.00
18	0.00	0.00	17.22	0.00	0.00	1.00
34	0.00	0.00	17.40	0.00	0.00	1.00
**(H2) Spatial proximity—all sheep**						
7	0.12	0.04	3.48	0.08	0.51	0.51
23	0.12	0.04	5.40	0.03	0.20	0.71
39	0.12	0.04	5.66	0.03	0.17	0.88
5	0.00	0.13	7.28	0.01	0.08	0.96
21	0.00	0.13	10.15	0.00	0.02	0.97
37	0.00	0.13	10.33	0.00	0.02	0.99
8	0.00	0.00	13.09	0.00	0.00	0.99
24	0.00	0.00	14.52	0.00	0.00	1.00
40	0.00	0.00	14.73	0.00	0.00	1.00
6	0.00	0.00	16.96	0.00	0.00	1.00
22	0.00	0.00	18.43	0.00	0.00	1.00
38	0.00	0.00	18.66	0.00	0.00	1.00
**(H3) Spatial proximity—non-family sheep**						
11	0.00	0.00	8.14	0.01	0.58	0.58
27	0.00	0.00	10.73	0.00	0.16	0.74
43	0.00	0.00	10.85	0.00	0.15	0.89
12	0.00	0.00	13.71	0.00	0.04	0.93
9	0.00	0.00	14.02	0.00	0.03	0.96
28	0.00	0.00	15.68	0.00	0.01	0.97
44	0.00	0.00	15.81	0.00	0.01	0.98
25	0.00	0.00	17.09	0.00	0.01	0.99
41	0.00	0.00	17.21	0.00	0.01	1.00
10	0.00	0.00	18.78	0.00	0.00	1.00
26	0.00	0.00	21.13	0.00	0.00	1.00
42	0.00	0.00	21.26	0.00	0.00	1.00
**(H4) Homogenous**						
14	0.00	0.00	8.14	0.01	0.60	0.60
30	0.00	0.00	10.55	0.00	0.18	0.78
46	0.00	0.00	10.85	0.00	0.15	0.93
15	0.00	0.00	13.71	0.00	0.04	0.97
31	0.00	0.00	15.68	0.00	0.01	0.98
47	0.00	0.00	15.81	0.00	0.01	1.00
13	0.00	0.00	18.78	0.00	0.00	1.00
29	0.00	0.00	21.13	0.00	0.00	1.00
45	0.00	0.00	21.26	0.00	0.00	1.00
**No social network**						
16	–	–	5.52	0.03	0.63	0.63
32	–	–	7.91	0.01	0.19	0.82
48	–	–	8.03	0.01	0.18	1.00

a Full descriptions of models can be found in Table 1.

^b^ LCI s = lower confidence interval for the model s parameter, PST = proportion of cases solved by social transmission, corresponding to the lower limit for s, ΔAICc = change in Akaike's information criterion corrected from the best performing model, H, hypothesis,—indicates where there was no s parameter in the model, as no social network was included.

^c^ Akaike Weight is the probability the model is the best Kullback-Leibler model in the set. Overall Akaike Weight for model i is $Akaike\;Weight_{i}=e^{\left(-1/2AICci\right)}/\sum je^{\left(-1/2AICcj\right)}$ where j is all models in the set, adjusted Akaike Weight is calculated within the model set, and cumulative Akaike Weight is the sum of the Akaike Weights within the model.

**Table 5:** Median model-averaged terms from the multi-network network-based diffusion analysis, for all social networks combined, and individually for the kinship network of family groups, spatial proximity network of all sheep, spatial proximity of non-family sheep, and the homogenous network.

	**Social network predictor**				
**Predictor**	**All social networks**	**(H1) Kinship network (family groups)**	**(H2) Spatial proximity—all sheep**	**(H3) Spatial proximity between non-family sheep**	**(H4) Homogenous network**
s—kinship (family groups) network	0.45	0.45	–	–	–
s—spatial proximity (all sheep)	0.00	–	1.43	–	–
s—spatial proximity (non-family sheep)	0.00	–	–	0.00	–
s—homogenous network	0.00	–	–	–	0.00
Asocial—lamb	−0.53	−0.53	−0.63	−0.44	−0.44
Asocial—twin	−19.72	−19.73	−19.20	−1.12	−1.12
Social—lamb	0.00	0.00	0.00	0.00	0.00
Social—twin	0.00	0.00	0.00	0.00	0.00

^a^ s = the relative strength of social transmission of lameness to the rate of asocial transmission of lameness, asocial = variable set to affect asocial transmission of lameness, social = variable set to affect social transmission of lameness.

^b^ Predictors (lamb and twin) are dummy coded, therefore the baseline for each is not being a lamb or not being a twin.

^c^ Full descriptions of all models are in Table 1.

Hypothesis 2 (lameness is transmitted through spatial co-location with any sheep) received some support with ∑ *Akaike Weight*_*overall*_ = 0.15 (Table 4, Figure 2). As in the family network, models fitted best when ewes and lambs had different baseline rates for acquisition of lameness (models 7, 23 and 39), and when this was accounted for, at least 4% of cases came from social transmission across the flock. The 4% estimate for the role of contact in disease transmission from the proximity sensors is different from the 15% estimate for the family network because these estimates are from two different network assumptions: family and close proximity estimated from the sensors.

Hypothesis 3 (lameness was transmitted through spatial co-location with non-family sheep) and hypothesis 4 (all sheep had equal risk of acquisition of lameness) received little support (Table 4, Figure 2), indicating it was unlikely that lameness was transmitted *via* spatial co-location with non-family sheep, or that all sheep had equal risk of acquisition of lameness. Models without a social network predictor (models 16, 32, and 48) all received little support (Table 4, Figure 2).

A constant baseline asocial rate of acquisition of lameness had most support (Table 4, Supplementary Table 6), indicating that the risk of acquisition of disease from the environment remained constant over the study period.

#### Linear mixed effects models of social contact and sheep characteristics and the environment

The two outcome variables used in the six linear mixed effect models for ewes, single and twin lambs were time per day in contact with family and time per day in contact with non-family sheep (Tables 6, 7). Lame single lambs and ewes spent less time in contact with non-family sheep than non-lame counterparts, a reduction of 0.70 (95% CI = 0.26–1.15) hours/day for single lambs and 0.20 (95% CI = 0.10–0.29) hours/day for ewes. Within family contact time did not change in single lambs and ewes but lame twins spent 0.49 (95% CI = 0.02–1.33) hours/day less with their family than non-lame twin lambs (Table 6).

**Table 6:** Linear mixed effects models for factors associated with daily time ewes and lambs spent with their family and other sheep.

		**Family contact**				**Out of family contact**			
**Variable**	***N* (%)**	**β_Full_**	**β_Conditional_**	**LCI**	**UCI**	**β_Full_**	**β_Conditional_**	**LCI**	**UCI**
*Ewes*
Ewe age (years) +1 unit	–	−0.01	−0.04	−0.17	0.09	**−0.09**	**−0.11**	**−0.21**	**−0.01**
Pasture size—0.69 ha	160 (30.8)	Ref.				Ref.			
Pasture size—1.34 ha	160 (30.8)	−0.32	−0.33	−0.68	0.03	−0.14	−0.14	−0.66	0.38
Pasture size—1.98 ha	**200 (38.5)**	**−0.62**	**−0.64**	**−0.96**	**−0.31**	**−0.89**	**−0.89**	**−1.37**	**−0.42**
Sheep gathered—no	360 (69.2)	Ref.				Ref.			
Sheep gathered—yes	160 (30.8)	0.08	0.20	−0.14	0.55	−0.20	−0.37	−0.82	0.09
Locomotion score—0–1	393 (75.6)	Ref.				Ref.			
Locomotion score ≥2	127 (24.4)	0.05	0.12	−0.09	0.32	**−0.20**	**−0.20**	**−0.29**	**−0.10**
Mean daily THI +1°C	–	−0.11	−0.14	−0.28	0.01	0.01	0.03	−0.19	0.25
Mean daily WCI +1°C	–	0.05	0.11	−0.15	0.37	−0.06	−0.14	−0.39	0.11
*Single lambs*
Pasture size—0.69 ha	160 (30.8)	Ref.				Ref.			
Pasture size—1.34 ha	160 (30.8)	−0.33	−0.34	−0.73	0.05	0.01	0.03	−1.09	1.16
Pasture size—1.98 ha	**200 (38.5)**	**−0.65**	**−0.68**	**−1.04**	**−0.31**	−0.33	−0.87	−1.91	0.18
Sheep gathered—no	360 (69.2)	Ref.				Ref.			
Sheep gathered—yes	160 (30.8)	0.05	0.16	−0.23	0.54	−0.16	−0.46	−1.72	0.79
Lamb age (days) +1 unit	–	−0.01	−0.02	−0.06	0.02	−0.02	−0.05	−0.14	0.05
Locomotion score—0–1	284 (84.0)	Ref.				Ref.			
Locomotion score ≥2	54 (16.0)	−0.06	−0.15	−0.42	0.12	**−0.70**	**−0.70**	**−1.15**	**−0.26**
Mean daily THI +1°C	–	−0.06	−0.10	−0.22	0.03	0.35	0.53	−0.06	1.11
Mean daily WCI +1°C	–	0.01	0.03	−0.23	0.29	−0.44	−0.73	−1.57	0.12
Sex—female	182 (53.8)	Ref.				Ref.			
Sex—male	**156 (46.2)**	**0.31**	**0.44**	**0.00**	**0.88**	−0.59	−0.97	−2.05	0.11
*Twin lambs*
Pasture size—0.69 ha	160 (30.8)	Ref.				Ref.			
Pasture size—1.34 ha	160 (30.8)	−0.43	−0.43	−0.79	−0.07	−0.34	−0.35	−1.42	0.72
Pasture size—1.98 ha	**200 (38.5)**	**−1.11**	**−1.11**	**−1.44**	**−0.79**	**−1.73**	**−1.79**	**−2.79**	**−0.80**
Sheep gathered—no	360 (69.2)	Ref.				Ref.			
Sheep gathered—yes	160 (30.8)	0.24	0.34	0.00	0.69	−0.18	−0.51	−1.57	0.55
Lamb age (days) +1 day	**–**	**−0.22**	**−0.22**	**−0.34**	**−0.11**	0.02	0.05	−0.07	0.18
Locomotion score—0–1	332 (91.2)	Ref.				Ref.			
Locomotion score ≥2	**32 (8.8)**	**−0.49**	**−0.67**	**−1.33**	**−0.02**	−0.02	−0.08	−0.69	0.52
Mean daily THI +1°C	**–**	**−0.17**	**−0.17**	**−0.32**	**−0.02**	0.28	0.46	−0.06	0.97
Mean daily WCI +1°C	–	0.06	0.14	−0.09	0.37	−0.44	−0.69	−1.44	0.07
Sex—female	169 (46.4)	Ref.				Ref.			
Sex—male	195 (53.6)	−0.40	−0.90	−2.23	0.42	0.18	0.58	−0.92	2.07

^a^ N, number of observations, β, coefficient, LCI, lower confidence interval, UCI, upper confidence interval, ha, hectare, WCI, wind-chill index (°C), THI, temperature-humidity index (°C). Variables are highlighted in bold where the confidence interval does not cross 0.

^b^ $\beta_{\text{Full}}$ is the model-averaged coefficient where it is assumed that the variable is included in every model, but in some models the coefficient (and its respective variance) is set to zero. β_Conditional_ the average coefficient over the models where the parameter is included.

^c^ The 95% confidence set of models is the model set is where the ∑*Akaike Weight* ≤ 0.95. In-family: 34 models for ewes, 72 for single lambs and 15 for twins, Out-of family: 15 models for ewes, 53 models for single lambs and 64 models for twins.

**Table 7:** Variable importance for variables in Table 6: factors associated with time ewes and lambs spent with their family and other sheep.

	**Variable importance (**∑***Akaike Weight*****)**					
	**In-family contact**			**Out of family contact**		
**Variable**	**Ewes**	**Singles**	**Twins**	**Ewes**	**Singles**	**Twins**
Ewe age (years) +1 year	0.29	–	–	0.78	–	–
Lamb age (days) + 1 day	–	0.34	1.00	–	0.35	0.32
Lamb sex (female, male)	–	0.70	0.45	–	0.61	0.31
Pasture size (0.69, 1.34, 1.98 ha)	0.97	0.96	1.00	1.00	0.38	0.97
Sheep gathered (no, yes)	0.41	0.33	0.71	0.54	0.34	0.36
Locomotion score (0–1, ≥2)	0.40	0.38	0.73	1.00	1.00	0.25
Mean daily THI (°C)	0.84	0.61	1.00	0.33	0.67	0.62
Mean daily WCI (°C)	0.46	0.36	0.42	0.43	0.60	0.64

^a^ Variable importance is the ∑*Akaike Weight* over all models including the variable. In-family: 34 models for ewes, 72 for single lambs and 15 for twins, Out-of family: 15 models for ewes, 53 models for single lambs and 64 models for twins.

^b^ THI, temperature-humidity index (°C), WCI, wind-chill index (°C), ha, hectare.

Ewes spent 0.11 (95% CI = 0.01–0.21) fewer hours/day with non-family sheep for each year of age; ewe age was not associated with time spent with their lambs. Twin lambs spent 0.22 (95% CI = 0.11–0.34) hours/day less with their family for each day of life (Table 6).

As pasture size increased ewes spent less time with family and non-family sheep (Table 6), and twin lambs spent less time with non-family sheep (Table 6), but there was no effect of pasture size on the time single lambs spent with non-family sheep. As the THI increased, twin lambs spent 0.17 (95% CI = 0.02–0.32) hours/day less with their family (Table 6).

## Discussion

This is the first study to investigate the role of social contact in the transmission of footrot in sheep. We used high-resolution contact data combined with phenotypic observations to investigate the role of the family network and spatial co-location networks in the acquisition of footrot. We demonstrate that social behavior within families is associated with acquisition of a proportion cases of lameness but that most transmission of footrot is asocial, that is, not associated with infection from specific sheep.

Asocial transmission of footrot has been reported previously in lambs born on straw bedding in pens with groups of ewes. Lambs were negative for *D. nodosus* at birth but within a few hours they had strains of *D. nodosus* on their interdigital skin not entirely consistent with those of their dam, indicating that even their first exposure to *D. nodosus* came from floor surfaces contaminated by several ewes rather than just their dam (45). Models fitted best when the underlying risk of acquisition of lameness from asocial transmission was constant over the 13 days (Table 4, Supplementary Table 6), this is consistent with pasture persistently contaminated with *D. nodosus*. Despite this, sheep did not have a homogenous risk of acquisition of lameness from the environment (Table 4). This is probably because pasture is not homogenously infectious, all areas of a field, whether frequently used by sheep or not, are heterogeneously contaminated with *D. nodosus* (5) and so it is “chance” whether a sheep “steps” into a contaminated area of the field. Further heterogeneity in acquisition of lameness might have come from sheep clustering by hedges for considerable time during wet weather (17, 19, 46, 47) and because sheep social behavior changed when they were given access to more (uncontaminated) pasture twice during the study (15). These managements are specific to this flock, but all flocks will have management actions that alter their behavior and might alter the risk of disease transmission.

Sheep with footrot contaminate the ground as they stand and walk and we hypothesize that sheep which spend considerable time spatially co-located with lame sheep are more likely to contact that contaminated ground and become diseased. Models using the family network (hypothesis 1) received more support than hypothesis 2 which considered spatial co-location between sheep (Table 4, Figure 2). It might be that it is the nature of very close contact only made within families, e.g., suckling, that brings sheep into sufficiently close contact for sufficient time for sheep to acquire infection from ground contaminated by a family member. Diseases with low infectiousness are particularly sensitive to the definition of a contact (48, 49) and the definition of contact we investigated (1–1.5 m and 20 s packets of time) in hypothesis 2, probably includes contacts that were never going to lead to transmission of footrot and so underestimated the role of close contact.

Even within families the total contact time influenced acquisition of lameness. Single lambs were more likely to become lame than twin lambs; they spent more time with their dam, possibly because they suckle for longer per day than twin lambs (50) and did not reduce contact time with their family with increasing age as did twin lambs, as in this study (Table 6) and Morgan and Arnold (13). Family networks might also be a risk for transmission of lameness because lame sheep changed their behavior and spent less time with non-family members (Table 6) which would contribute to more within family, social, spread of footrot.

Risks from family networks could also be explained by other factors such as genetic susceptibility to footrot and all sheep in the current study were related to greater or lesser extent. Heritability to footrot is low (0.1) (10) and it is unlikely that heritability explains all the transmission of footrot within families. Families also group together and it might be hypothesized that certain families spent more time in “high risk” areas of the field, however, as Clifton et al. (5) report, *D. nodosus* is found across pasture—there are no “high risk” areas in fields. Overall, we conclude that it is the time that families spend together that increases the risk of transmission of footrot.

The risk of transmission of *D. nodosus* within families was bidirectional, sometimes ewes were lame before their lambs, and vice versa, which was also reported in another longitudinal study (30). In both that study and the current study, lambs were not observed from birth and so social behavior and the first occurrence of footrot might have been missed, since the incubation period for footrot is 7–14 days (51, 52). One might hypothesize that since lambs are born without *D. nodosus* infection (45), the first lamb in a flock infected must have been infected from the ewes, but after that one lamb is infected, infection is bi-directional between ewes and lambs. A longer study observing lambs from birth would be needed to investigate the time to first infection and how changes in lamb behavior with age change the risk of routes of transmission.

Whilst a small proportion of all transmission was social, the finding is important because disease control depends on understanding all routes of transmission. In a flock where ewes and lambs are penned separately all transmission would be within family (52). However, it is useful to know that even in a field where ewes and lambs mix freely some spread of disease arises within families. Isolation of lame sheep reduces onward spread of disease so from our study we can conclude that separation of whole families once one is lame with footrot could protect the rest of the flock since it is likely that another family member is incubating disease. There was little evidence of social transmission of footrot outside families (Table 4). Our study suggests that short contact times and many combinations of pairs of contacts between non-family sheep are not sufficient for transmission. In addition, lame sheep spent less time with non-family sheep than non-lame sheep (Table 6), which could also be why lameness was not transmitted as a result of social contact with non-family sheep.

A limitation of the study is that we used lameness to define infectiousness and so sheep with mild ID that were not lame would have been categorized as non-infectious: we do not know if these sheep are infectious, but they have a lower load of *D. nodosus* than diseased sheep (33). There were sheep with ID at the end of our study that were not lame (Supplementary Figures 1, 2, Supplementary Table 5). If those sheep were infectious then they are missing from the order of acquisition, which would reduce the power of NBDA to detect a social effect (53). However, it would have perturbed sheep behavior and so affected our hypotheses to examine sheep daily for signs of mild lesions. In addition, sheep would have been incubating footrot, but not lame, at the start of the study, and so the origin of their infection was not known and would have been “asocial” in our analyses. We removed families from the analysis if one member of the family had incomplete data to avoid inflated probabilities of association with non-family sheep. However, data on as few as 30% of individuals in a social network can give an accurate representation of their position in the network (54).

## Conclusions

Our study illustrates that high-resolution contact data together with phenotypic observation provides insights into the role of social networks in transmission of *D. nodosus* within a sheep flock. While most transmission of *D. nodosus* occurs asocially in a flock of ewes with young lambs, at least 15% of cases occur from within family transmission. We conclude that this is because sheep spend more time with their family than with non-family sheep, and because lame sheep reduce the time they spend with non-family sheep. Further evidence that duration of close contact is important is that single lambs were more likely to become lame than twin lambs, and spent more time with their dam than did twin lambs.

## Data availability statement

The raw data supporting the conclusions of this article will be made available by the authors, without undue reservation.

## Ethics statement

The animal study was reviewed and approved by University of Exeter-eCLESPsy000541.

## Author contributions

KL analyzed the data and wrote the manuscript draft. EP, JL, and KL collected the farm data. LO and CC processed the proximity sensor data. LG, DC, and JL conceived the study design. All authors contributed to the article, reviewed, and approved the submitted version.

## Funding

KL was supported by a BBSRC MIBTP iCase Studentship (training grant number BB/M01116X/1) with the Agriculture and Horticulture Development Board. EP was supported by a BBSRC Studentship (training grant number BB/M009122/1).

## Conflict of interest

Author JL is a director of ActivInsights Ltd. The remaining authors declare that the research was conducted in the absence of any commercial or financial relationships that could be construed as a potential conflict of interest.

## Publisher's note

All claims expressed in this article are solely those of the authors and do not necessarily represent those of their affiliated organizations, or those of the publisher, the editors and the reviewers. Any product that may be evaluated in this article, or claim that may be made by its manufacturer, is not guaranteed or endorsed by the publisher.
