# Supplementary material for: Social behaviour and transmission of lameness in a flock of ewes and lambs
Source: Front Vet Sci. 2022 Dec 1;9:1027020. doi: 10.3389/fvets.2022.1027020 (PMC9753574; doi:10.3389/fvets.2022.1027020)
Supplement: Supplementary file 1 [file Data_Sheet_1.docx]

Supplementary Material

Supplementary Table 1 Descriptive summary of mean daily weather-related variables (temperature, humidity, temperature-humidity index, wind-chill index, total rainfall and sunlight hours) recorded by the on-farm Davis Vantage Pro2 Plus weather station

| **Date** | **Mean temperature (°C)** | **Mean humidity (°C)** | **Mean THI (°C)** | **Mean WCI (°C)** | **Mean windspeed (mph)** | **Total rainfall (cm)** | **Sunlight hours (hours)** |
| --- | --- | --- | --- | --- | --- | --- | --- |
| 2019-10-01 | 13.8 | 95.8 | 56.8 | 10.8 | 5.5 | 0.8 | 11.3 |
| 2019-10-02 | 9.2 | 84.5 | 49.3 | 6.3 | 3.5 | 0.0 | 11.2 |
| 2019-10-03 | 9.8 | 93.3 | 50.0 | 6.7 | 4.2 | 0.4 | 11.2 |
| 2019-10-04 | 12.1 | 93.2 | 54.0 | 9.3 | 4.4 | 0.2 | 11.1 |
| 2019-10-05 | 13.0 | 96.7 | 55.4 | 11.3 | 2.4 | 0.9 | 11.0 |
| 2019-10-06 | 11.7 | 91.0 | 53.2 | 8.6 | 4.6 | 0.2 | 11.0 |
| 2019-10-07 | 11.6 | 97.7 | 52.9 | 8.7 | 4.1 | 0.3 | 10.9 |
| 2019-10-08 | 11.4 | 91.3 | 52.8 | 8.5 | 4.2 | 0.1 | 10.8 |
| 2019-10-09 | 9.4 | 93.5 | 49.2 | 6.3 | 3.9 | 0.5 | 10.8 |
| 2019-10-10 | 11.4 | 94.1 | 52.7 | 7.9 | 5.6 | 0.1 | 10.7 |
| 2019-10-11 | 12.6 | 98.2 | 54.7 | 8.8 | 7.4 | 2.6 | 10.7 |
| 2019-10-12 | 10.1 | 97.8 | 50.3 | 8.7 | 1.7 | 1.0 | 10.6 |
| 2019-10-13 | 10.4 | 95.9 | 50.8 | 7.9 | 3.1 | 1.3 | 10.5 |
| 2019-10-14 | 10.4 | 98.5 | 50.9 | 7.7 | 3.5 | 1.0 | 10.5 |
| 2019-10-15 | 10.9 | 95.3 | 51.8 | 9.0 | 2.3 | 0.1 | 10.4 |

1. THI = temperature humidity index (°C), WCI = wind chill index (°C), mph = miles per hour, cm = centimetre

Supplementary Table 2 Criteria from the Kaler et al., 2009 locomotion scoring system for sheep

| **Criteria - all required for score** | **Locomotion score** | | | | | | |
| --- | --- | --- | --- | --- | --- | --- | --- |
|  | **0** | **1** | **2** | **3** | **4** | **5** | **6** |
| Bears weight evenly on all four feet |  |  |  |  |  |  |  |
| Uneven posture, but no clear shortening of stride |  |  |  |  |  |  |  |
| Short stride on one leg compared to others |  |  |  |  |  |  |  |
| Visible nodding of head in time with short stride |  |  |  |  |  |  |  |
| Excessive flicking of head, more than nodding, in time with short stride |  |  |  |  |  |  |  |
| Not weight bearing on affected limb when standing |  |  |  |  |  |  |  |
| Discomfort when moving |  |  |  |  |  |  |  |
| Not weight bearing on affected limb when moving |  |  |  |  |  |  |  |
| Extreme difficulty rising |  |  |  |  |  |  |  |
| Reluctant to move once standing |  |  |  |  |  |  |  |
| More than one limb affected |  |  |  |  |  |  |  |
| Will not stand or move |  |  |  |  |  |  |  |

1. Shaded squares indicate where the criterion is required

Supplementary Table 3 The adapted scoring system from Moore et al., 2005 for interdigital dermatitis and severe footrot lesions in ewes and lambs

| **Foot lesion** | **Criteria** |
| --- | --- |
| Interdigital dermatitis |  |
| 0 | Clean interdigital foot with no lesions |
| 1 | Slight interdigital dermatitis, partial loss of hair, slight redness but dry |
| 2 | Slight interdigital dermatitis, partial/complete loss of hair, redness, pasty scum (<10% of interdigital area affected) |
| 3 | Moderate interdigital dermatitis, partial/complete loss of hair, redness, pasty scum (10-50% of interdigital area affected) |
| 4 | Severe interdigital dermatitis, partial/complete loss of hair, redness, pasty scum (>50% of interdigital area affected) |
| 4+ | The interdigital space was hairless, reddish, swollen, cracked or ulcerated – evidence there was an inflammatory process. No under-running of the hoof wall. |
| Severe footrot |  |
| 0 | No under-running of the wall or sole of the digit |
| 1 | Under-running of the horn on the wall and/or sole of the digit |
| 2 | Extensive under-running and detachment of the horn involving the sole and wall of the digit |

Supplementary Figure 1 Visual representations of the social network hypotheses tested within the NBDA for four example family groups


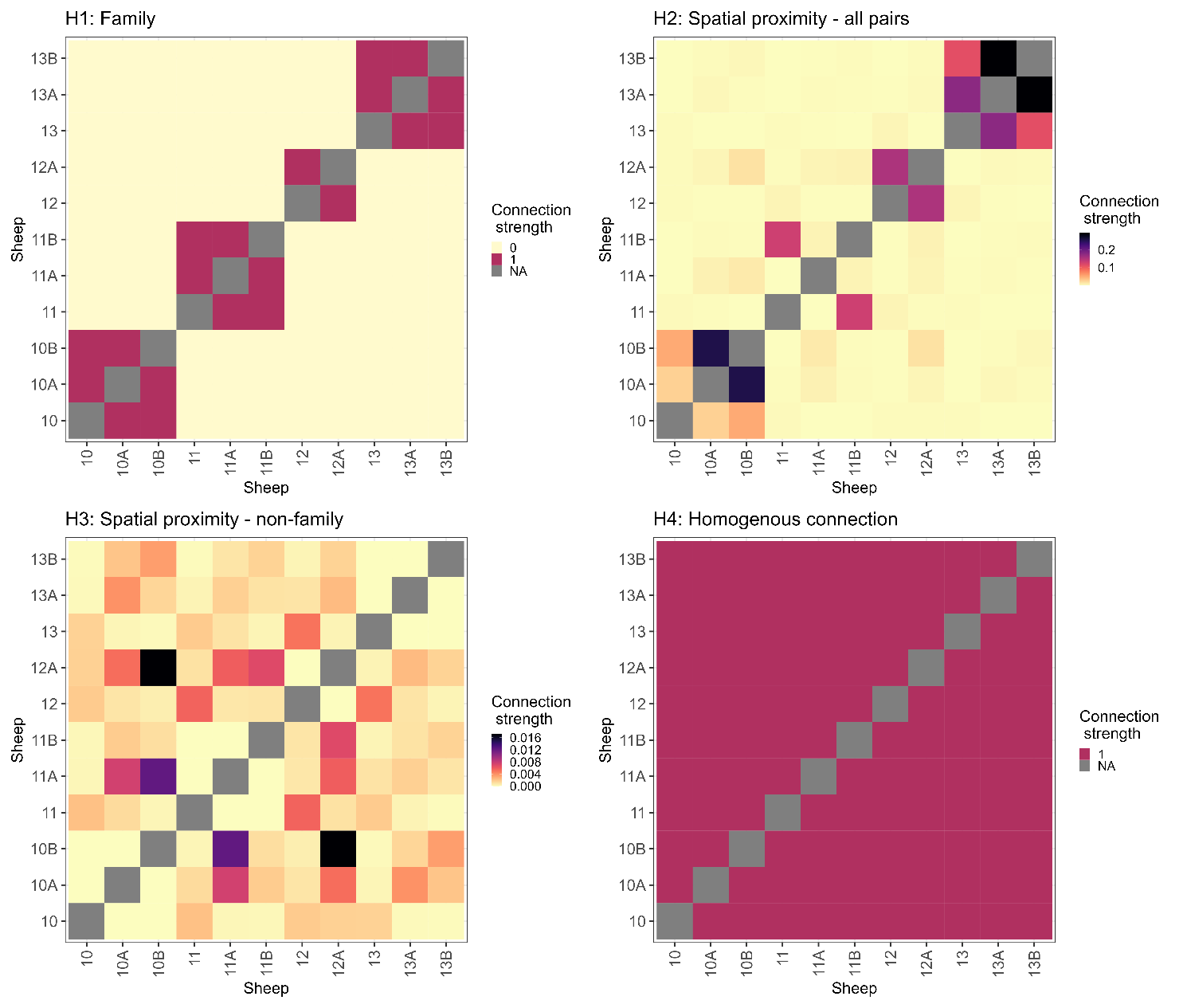


Each sheep is represented on both the x and y axis, the diagonal (grey) is each sheep with itself. Ewes are represented with a number, and their lambs with the same number and a letter.

**Association indexes between pairs of sheep**

Association indexes were calculated as in Ozella et al., 2022.

Supplementary Table 4 Association indexes for dyadic associations between related and non-related sheep from the static association network calculated over the 13-day study period.

| **Dyad type** | **Mean** | **Median** | **SD** | **Min** | **Max** |
| --- | --- | --- | --- | --- | --- |
| Ewe–offspring (family group) | 0.183 | 0.169 | 0.123 | 0.000 | 0.494 |
| Non-family group sheep | 0.003 | 0.001 | 0.008 | 0.000 | 0.334 |
| Ewe–non-kin sheep | 0.002 | 0.001 | 0.005 | 0.000 | 0.212 |
| Single lamb–mother | 0.233 | 0.210 | 0.100 | 0.062 | 0.494 |
| Single lamb–non-kin sheep | 0.003 | 0.002 | 0.004 | 0.000 | 0.032 |
| Twin lamb– mother | 0.088 | 0.078 | 0.049 | 0.000 | 0.180 |
| Twin lamb– non-kin sheep | 0.003 | 0.001 | 0.010 | 0.000 | 0.334 |
| Twin lamb– other twin | 0.282 | 0.297 | 0.133 | 0.002 | 0.472 |

1. Min = minimum, max = maximum, SD = standard deviation

Prevalence of lameness in ewes and lambs in October

Overall, the daily prevalence of lameness ranged from 13.6% to 20.4% from the 1^st^ to the 15^th^ October 2019. From the 94 sheep with working sensors, the daily prevalence of lameness was 14.9% on day 0, and 17.0% on day 14, with a maximum of 21.3% on day 5.

a) Ewes


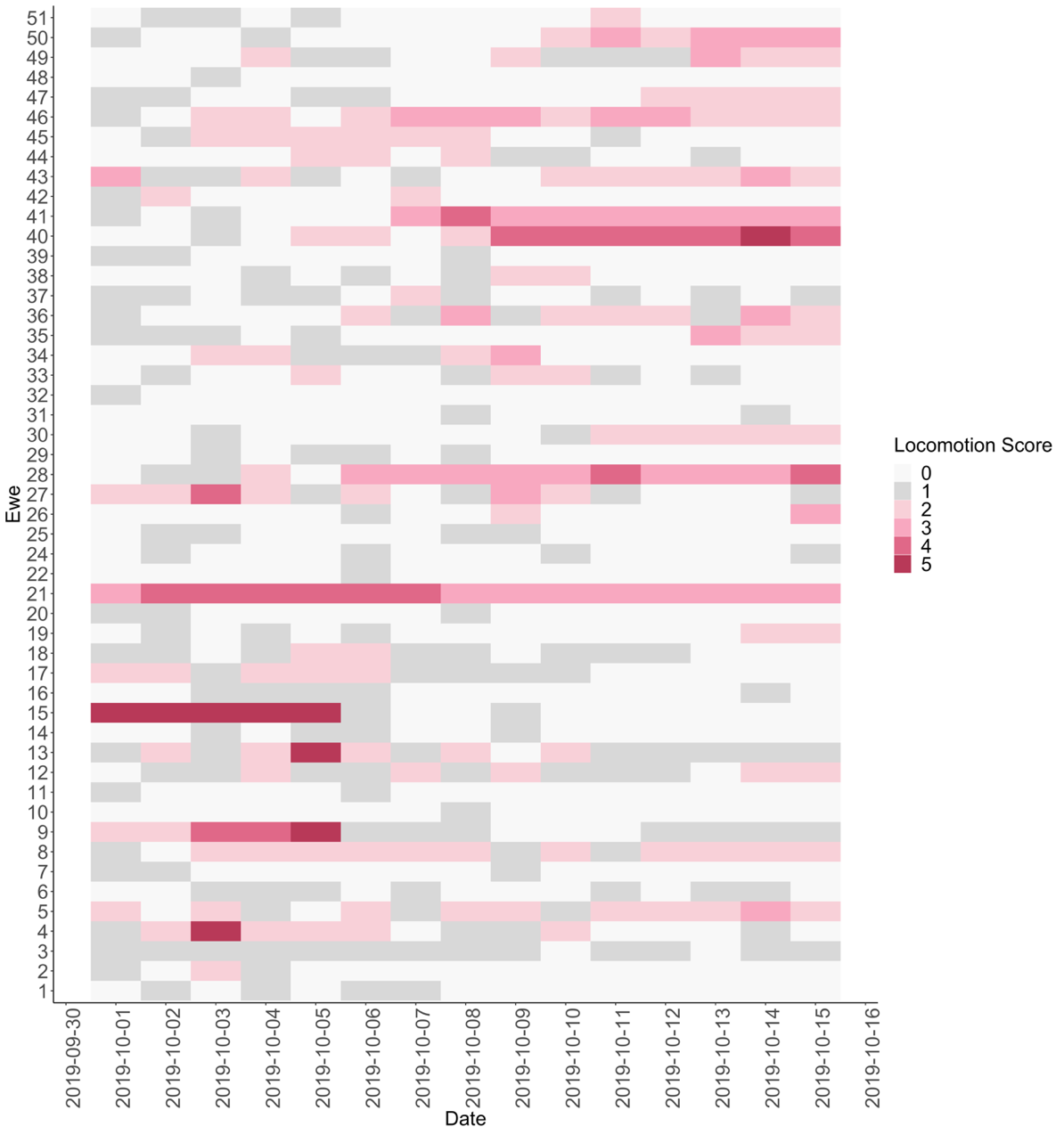


Supplementary Figure 2 Daily locomotion scores for 50 ewes from 1st-15th October 2019. Sound sheep (locomotion score 0 or 1) are shown in white or grey, respectively, then lame sheep (locomotion score of ≥2) are shown in pink-red.

b) Lambs


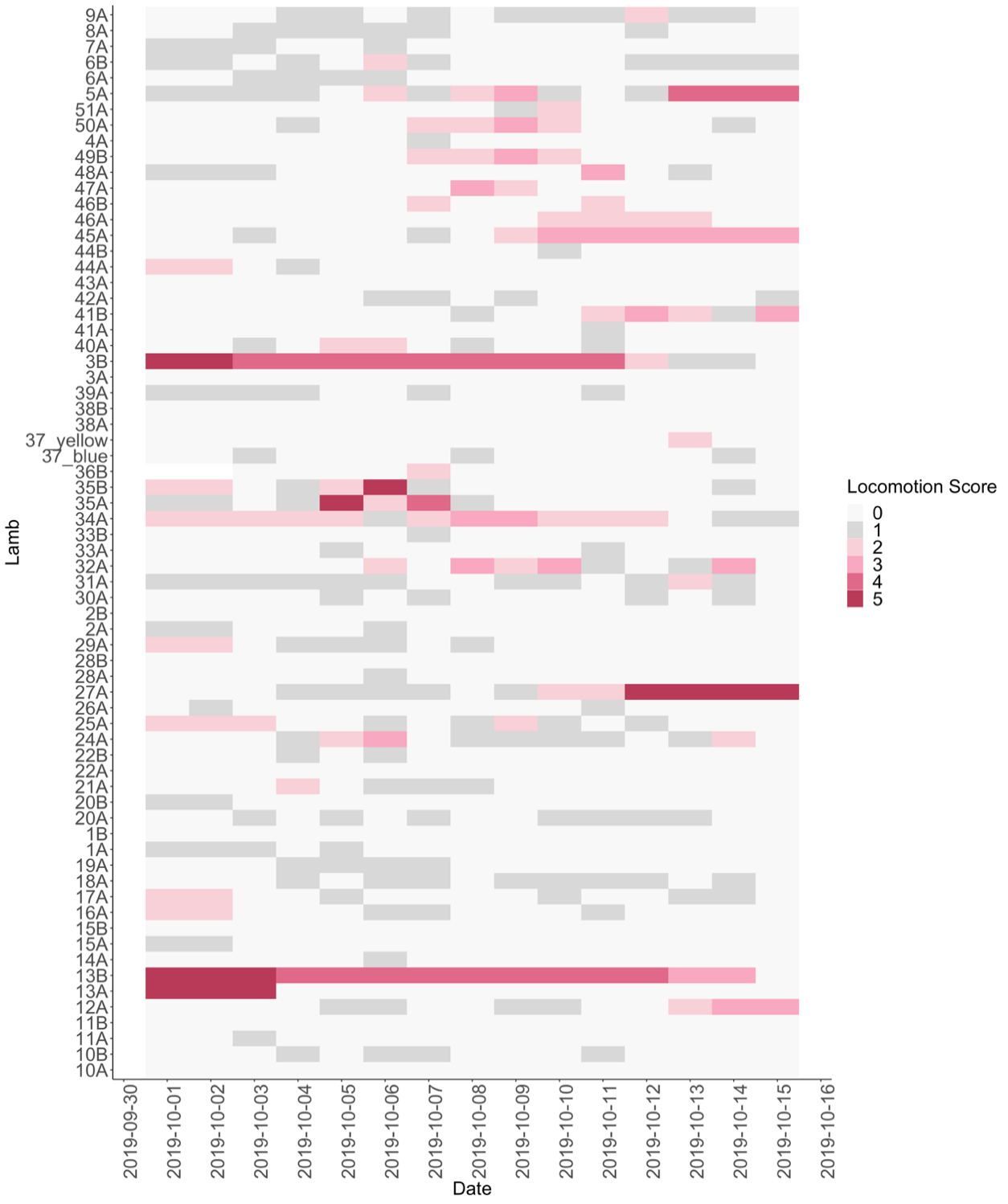


Supplementary Figure 3 Daily locomotion scores for 68 lambs from 1st-15th October 2019. Sound (locomotion score 0 or 1) are shown in white or grey, respectively, then lame sheep (locomotion score of ≥2) are shown in pink to red.

Causes of lameness

On the final day of the trial, 37.0% of lambs and 30.0% of ewes had an ID lesion and only one ewe showed any evidence of SFR with the ID. White line abscesses and fibroma were seen in ewes but not lambs. Some sheep (4 ewes and 1 lamb) had both an infectious and non-infectious cause of lameness. Non-infectious causes of lameness for lambs included granuloma (1 lamb) and a possible shoulder injury (1 lamb).

Supplementary Table 5 Number and percentage of ewes and lambs with each potential cause of lameness identified at the end of the study from the 94 sheep with 13 days of midnight-midnight contact data

| **Potential cause of lameness** | | **Ewes** | | **Lambs** | | |  |
| --- | --- | --- | --- | --- | --- | --- | --- |
|  |  | **N** | **%** | | **N** | **%** | |
| *Infectious foot lesions* |  |  |  | |  |  | |
| Interdigital dermatitis score | 0 | 28 | 70.0 | | 34 | 63.0 | |
|  | 1 | 0 | 0.0 | | 0 | 0.0 | |
|  | 2 | 1 | 2.5 | | 0 | 0.0 | |
|  | 3 | 5 | 12.5 | | 10 | 18.5 | |
|  | ≥4 | 6 | 12.0 | | 10 | 18.5 | |
| Severe footrot score | 0 | 39 | 97.5 | | 54 | 100.0 | |
|  | 1 | 1 | 2.5 | | 0 | 0.0 | |
|  | ≥2 | 0 | 0.0 | | 0 | 0.0 | |
| *Non-infectious foot lesions* | | |  | |  |  | |
| White line disease | No | 33 | 82.5 | | 0 | 0.0 | |
|  | Yes | 7 | 17.5 | | 54 | 100.0 | |
| Fibroma | No | 33 | 82.5 | | 0 | 0.0 | |
|  | Yes | 7 | 17.5 | | 54 | 100.0 | |
| Granuloma | No | 40 | 100.0 | | 53 | 98.1 | |
|  | Yes | 0 | 0.0 | | 1 | 1.9 | |
| Heel ulcer | No | 39 | 98.0 | | 54 | 100.0 | |
|  | Yes | 1 | 2.0 | | 0 | 0.0 | |
| Broken claw at toe | No | 39 | 98.0 | | 54 | 100.0 | |
|  | Yes | 1 | 2.0 | | 0 | 0.0 | |
| Uneven claw size | No | 39 | 98.0 | | 54 | 100.0 | |
|  | Yes | 1 | 2.0 | | 0 | 0.0 | |
| Suspected shoulder injury | No | 40 | 100.0 | | 53 | 98.1 | |
|  | Yes | 0 | 0.0 | | 1 | 1.9 | |

1. N = number of sheep, % = percentage

**Testing assumptions about the baseline rate of acquisition of lameness in the network-based diffusion analysis**

The constant baseline models received 3.16 times as much support as the Gamma baseline models, and 2.86 times as much support as the Weibull baseline models (Supplementary Table 6), indicating that assuming the rate of acquisition of lameness in the absence of social transmission remains constant over time fitted the data better than allowing for increases or decreases in the asocial rate of acquisition over time according to either a Gamma or Weibull distribution.

Supplementary Table 6 Relative support for the models across the different baseline rate of acquisition.

| **Baseline** | **Support** | **Number of models** |
| --- | --- | --- |
| Constant | 0.60 | 16 |
| Gamma | 0.19 | 16 |
| Weibull | 0.21 | 16 |

Support is calculated as the sum of Akaike Weights for all models (detailed in Table 1 in the main manuscript) containing the relevant baseline assumption.

**References**

KALER, J., WASSINK, G. J. & GREEN, L. E. 2009. The inter- and intra-observer reliability of a locomotion scoring scale for sheep. *Vet Journal,* 180**,** 189-94.

MOORE, L. J., WASSINK, G. J., GREEN, L. E. & GROGONO-THOMAS, R. 2005. The detection and characterisation of Dichelobacter nodosus from cases of ovine footrot in England and Wales. *Vet Microbiol,* 108**,** 57-67.

OZELLA, L., PRICE, E., LANGFORD, J., LEWIS, K. E., CATTUTO, C. & CROFT, D. P. 2022. Association networks and social temporal dynamics in ewes and lambs. *Applied Animal Behaviour Science,* 246**,** 105515.
